# A Nuclear Singlet Lifetime of More than One Hour in Room-Temperature Solution**

**DOI:** 10.1002/anie.201411978

**Published:** 2015-02-04

**Authors:** Gabriele Stevanato, Joseph T Hill-Cousins, Pär Håkansson, Soumya Singha Roy, Lynda J Brown, Richard C D Brown, Giuseppe Pileio, Malcolm H Levitt

**Affiliations:** School of Chemistry, University of Southampton University Road, Southampton, SO17 1BJ (UK)

**Keywords:** long-lived spin states, NMR spectroscopy, nuclear spin relaxation, singlet states

## Abstract

Nuclear magnetic resonance (NMR) and magnetic resonance imaging (MRI) are supremely important techniques with numerous applications in almost all branches of science. However, until recently, NMR methodology was limited by the time constant *T*_1_ for the decay of nuclear spin magnetization through contact with the thermal molecular environment. Long-lived states, which are correlated quantum states of multiple nuclei, have decay time constants that may exceed *T*_1_ by large factors. Here we demonstrate a nuclear long-lived state comprising two ^13^C nuclei with a lifetime exceeding one hour in room-temperature solution, which is around 50 times longer than *T*_1_. This behavior is well-predicted by a combination of quantum theory, molecular dynamics, and quantum chemistry. Such ultra-long-lived states are expected to be useful for the transport and application of nuclear hyperpolarization, which leads to NMR and MRI signals enhanced by up to five orders of magnitude.

Many magnetic resonance experiments would benefit from the ability to maintain nuclear spin order for a long time under ambient conditions. Currently, nuclear spin order lifetimes exceeding one hour have only been achieved in the gas phase.[1] It is much more difficult to achieve long-term storage of nuclear spin order in solution.

Extended spin order lifetimes may be achieved using long-lived nuclear spin states, which are spin configurations of coupled magnetic nuclei which exhibit extended relaxation times, often exceeding *T*_1_ by an order of magnitude.[2–14] Long-lived states have particular promise in the context of hyperpolarized magnetic resonance,[15] where the lifetime of the large signal enhancement is normally determined by *T*_1_. The hyperpolarization lifetime may be extended by using long-lived states.[4], [5]

The simplest form of long-lived nuclear spin states involves a pair of spin-1/2 nuclei, and is called singlet order.[2], [9] The fundamental requisites for a molecule to display long-lived singlet order are well-known: the molecule must contain a pair of spin-1/2 nuclei, remote from other spins in order to minimize the dipolar and scalar coupling relaxation. The spin pair must be magnetically inequivalent in order to allow access to the long-lived state. So far, the longest reported nuclear singlet lifetimes are 26 minutes[3] for the ^15^N pair in ^15^N_2_O and 16 minutes for the ^13^C pair in some acetylene derivatives[10] (both measured in low magnetic field). Systems exhibiting even longer-lived states are of great potential interest for molecular imaging applications, particularly if the molecular system is amenable to functionalization.

We have now studied the following naphthalene derivative: 1,2,3,4,5,6,8-heptakis([D_3_]methoxy)-7-(([D_7_]propan-2-yl)oxy)-naphthalene (compound **I**). ^13^C labeling of **I** at both positions 4a and 8a (Figure 1 a) generates a ^13^C_2_ spin pair near the center of the molecule. The synthetic route to ^13^C_2_-**I** is reported elsewhere.

**Figure 1:**
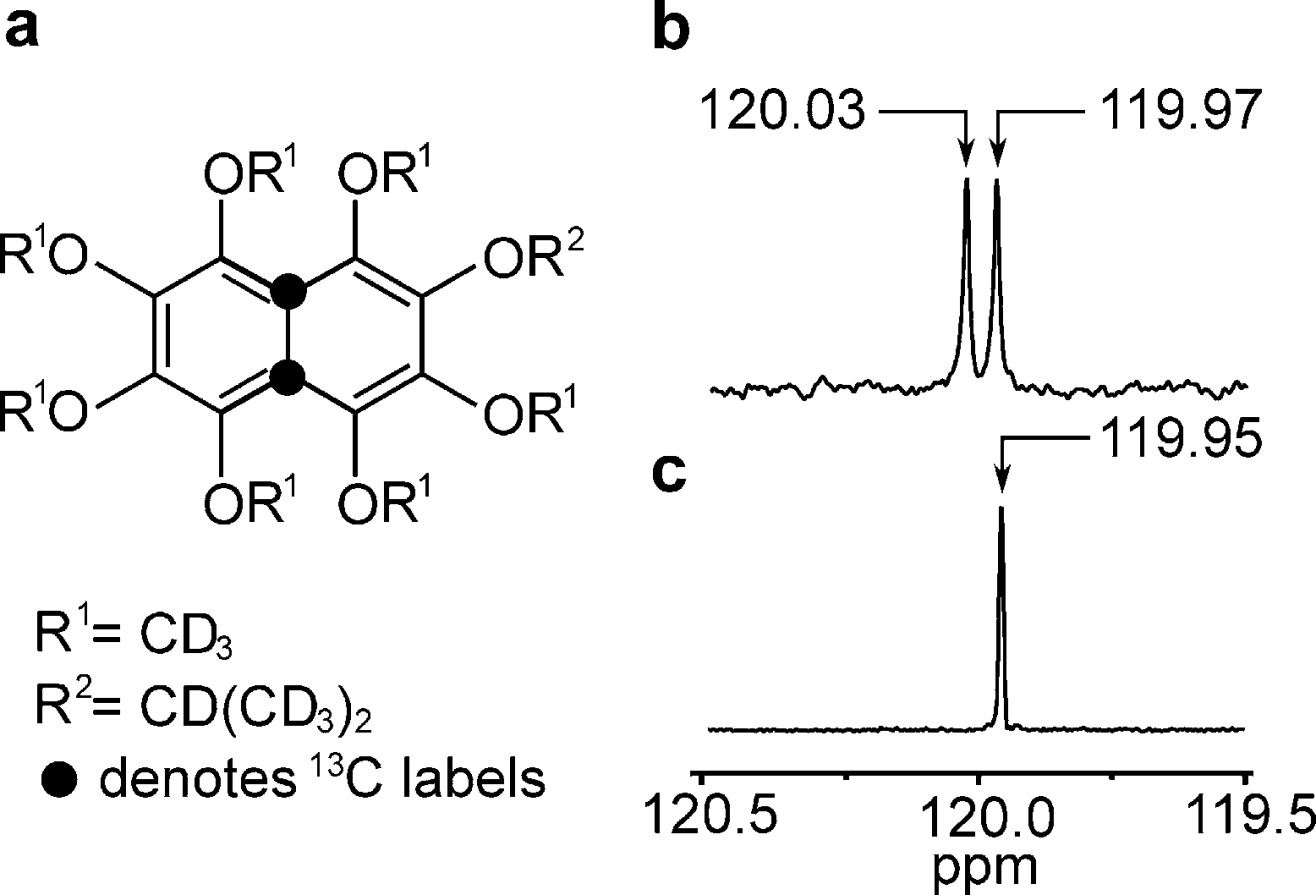
a) Molecular structure of the naphthalene derivative ^13^C_2_-I. Black filled circles indicate the ^13^C_2_ labeling sites. b) ^13^C spectrum of I in [D_6_]acetone solution, acquired at 9.4 T with 512 transients. The two peaks separated by 0.06 ppm indicate the resonances of the inequivalent ^13^C nuclei. c) The ^13^C spectrum of ^13^C_2_-I acquired at 9.4 T with a single transient shows a single peak with a full-width-at-half-height of 1.5 Hz.

The asymmetric deuterated side-chains generate a small chemical shift difference between the ^13^C sites of Δ*δ*≈0.06±0.02 ppm, as shown by the natural abundance ^13^C spectrum of unlabeled **I** shown in Figure 1 b. The small chemical shift difference is required to access the long-lived singlet state through radiofrequency pulse sequences.[6], [7] The ^13^C spectrum of ^13^C_2_-**I** shows a single narrow peak (Figure 1 c). The theoretical splitting of Δ*δ*^2^*ν*_a_^2^/2 *J*_CC_≅0.3 Hz for the strongly coupled AB ^13^C_2_ spin pair, where *ν*_a_ is the ^13^C resonance frequency, is not resolved at this field. The 0.05 ppm difference between the mean chemical shift of the natural abundance material and the ^13^C_2_-labelled compound is attributed to an isotope shift.[16]

Figure 2 a shows experimental singlet decay curves measured at 9.4 T (gray triangles) and 0.4 T (black circles) with the method described in the Supporting Information. As in previous measurements[3] these data exhibit a rapid initial drop due to equilibration of the triplet states, followed by a much slower decay due to singlet relaxation. In the current case the timescales are so well separated that the value of *T*_S_ is readily estimated by fitting the data to an exponential decay, omitting the first point.

**Figure 2:**
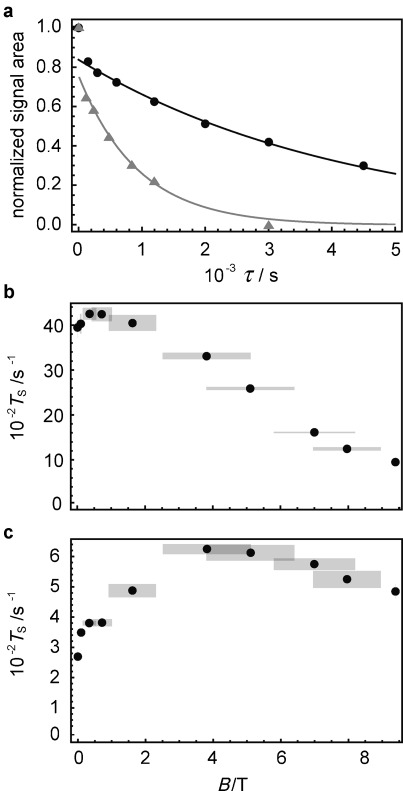
a) Experimental decay curves for 0.1 m ^13^C_2_-I dissolved in degassed [D_6_]acetone obtained using the pulse sequence in the Supporting Information for magnetic fields *B*=9.39 T (gray triangles) and 0.4 T (black circles). Experimental points have been normalized to the value of the first point. Solid lines are fits to exponential decays, ignoring the first point at *τ*=1 s. Experimental field dependence of *T*_S_ for ^13^C_2_-I in [D_6_]acetone for b) a degassed sample, and c) a non-degassed sample with [O_2_]≈2 mm. Gray rectangles represent the confidence intervals.

The measured values of *T*_S_ and *T*_1_ are reported in Table 1 and Figure 2 as a function of magnetic field. A range of fields was accessed by transporting the sample along the magnet bore during the pulse sequence (see the Supporting Information). This method generates wide confidence limits for intermediate magnetic field values, as indicated in the plots.

**Table 1:** Singlet (*T*_S_) and magnetization (*T*_1_) decay time for ^13^C_2_-I in [D_6_]acetone as a function of magnetic field *B*

*B* [T]	*T*_S_ [s]^[a]^	*T*_1_ [s]^[a]^	*T*_S_ [s]^[b]^	*T*_1_ [s]^[b]^
0.0020±10^−4^	3950±220	78±3	270±20	33±1
0.10±0.02	4030±220	72±2	348±8	37±2
0.4±0.2	4250±130	73±2	380±8	40±1
0.7±0.3	4240±150	76±2	381±12	46±1
1.6±0.7	4050±180	73±2	487±22	48±2
3.8±1.3	3310±70	55±2	624±17	37±1
5.1±1.3	2590±30	43±1	611±26	29±1
7.0±1.2	1610±20	29±1	574±19	22±1
8.0±1.0	1240±40	24±1	524±28	19±1
9.39	950±60	19±1	485±23	18±1

[a] Degassed sample; [b] non-degassed sample with [O_2_] about 2 mm.

For a degassed sample, the singlet decay time constant *T*_S_ exceeds one hour at 2 mT and becomes even longer as the field is increased, reaching a maximum of 4250±130 s at about 0.4 T. A further increase in the field reduces the singlet lifetime. The ratio *T*_S_/*T*_1_ is about 50 over a wide range of fields.

The relaxation times are significantly shorter when the sample is not degassed, leading to an estimated concentration of 2 mm for dissolved paramagnetic oxygen (Figure 2 c and Table 1). Nevertheless the singlet relaxation time still exceeds 10 minutes for fields around 4 T. The O_2_ concentration was estimated from the known Henry coefficient in acetone[17] and the partial pressure of O_2_ at 293.15 K (19 kPa).

We have found that standard analytical treatments of nuclear singlet relaxation, which are based on rigid molecular tumbling models,[10–13] fail to describe the singlet relaxation in this case. The very small singlet relaxation rates are strongly influenced by internal molecular flexibility, despite the relatively rigidity of the naphthalene core. An improved understanding of nuclear singlet relaxation is provided by a computational approach based on molecular dynamics and quantum chemistry, which is described in detail elsewhere. This involves QM/MM dynamics simulations of the molecule in solution followed by interpolation of the spin interaction tensors and correlation time estimations (see the Supporting Information).

The computational predictions for several mechanistic contributions to singlet relaxation in ^13^C_2_-**I** are summarized in Figure 3, which also shows a comparison with experiment. The relevant mechanisms are as follows:

**Figure 3:**
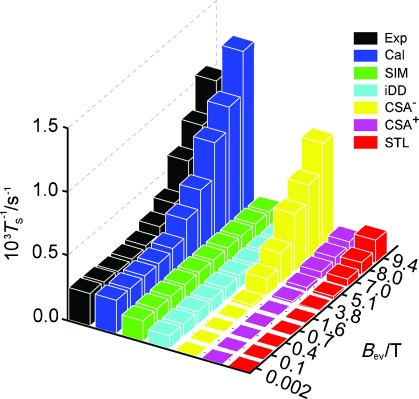
Experimental (Exp) and calculated (Cal) singlet state decay rates (*T*_S_^−1^) for ^13^C_2_-I. The experimental results are for a degassed solution in [D_6_]acetone. The individual simulated contributions of spin-internal-motion (SIM), intramolecular dipolar (iDD), symmetric (CSA^+^), and antisymmetric (CSA^−^) chemical shift anisotropy, and singlet–triplet leakage (STL) relaxation are shown.

1. Chemical shift anisotropy (CSA). The difference in the instantaneous CSA tensors can induce relaxation of singlet states.[10], [11] The Cartesian form of the shielding tensor can be decomposed into a scalar term, a symmetric part (CSA^+^), and antisymmetric part (CSA^−^), which both cause relaxation.[10], [11], [18] The local inversion geometry around the spin pair causes the two CSA tensors to be almost identical in the equilibrium structure[19] so that their difference vanishes. Nevertheless, the computational analysis shows that there is significant CSA-driven singlet relaxation in ^13^C_2_-**I**. Simulations show that transient fluctuations in the molecular geometry, and asymmetrical molecular perturbations, are responsible. As shown in Figure 3, the antisymmetric CSA relaxation mechanism dominates for singlet relaxation in ^13^C_2_-**I** in high magnetic field.
2. Spin-rotation (SR) and Spin-internal-motion (SIM). The spin-rotation interaction describes the coupling of the nuclei to fluctuating local magnetic fields caused by the overall rotational motion of the molecule.[3], [11] Coupling to the overall molecular tumbling gives a 10^−6^ s^−1^ contribution to *T*_S_^−1^ and is thus negligible in the case of ^13^C_2_-**I**. However, we have identified an important variant of this mechanism, which we call spin-internal-motion (SIM) relaxation. This is associated with fluctuating magnetic fields caused by some internal vibrational motions (typically rotations, bends, and twists). In the case of a rotating moiety such as a CH_3_ group, this mechanism corresponds to internal spin-rotation.[20] Our investigations show that SIM is significant for flexible molecules even if there are no obvious rotating parts. Figure 3 shows that SIM provides the strongest singlet relaxation mechanism for ^13^C_2_-**I** in low magnetic fields, despite the relatively high rigidity of the molecular core.
3. Intramolecular dipole–dipole relaxation (iDD). The motional modulation of the dipole-dipole couplings between the spin pair and other magnetic nuclei within the same molecule cause singlet relaxation.[11], [13] This is only a small contribution in ^13^C_2_-**I** as all other spins (^2^ H) have low γ and are far from the spin pair. This mechanism is small but significant for ^13^C_2_-**I** in low magnetic field.
4. Singlet–triplet leakage (STL).[10], [11] This effect is due to symmetry-breaking coherent spin interactions that mix the singlet state with triplet states. In the case of ^13^C_2_-**I**, the symmetry-breaking is provided by the small isotropic shift difference, and contributes minimally to singlet relaxation, except at high magnetic fields.
5. Intermolecular relaxation. Nuclear singlet relaxation from dissolved paramagnetic species may be significant.[14] The data on a non-degassed sample display the effect of dissolved molecular oxygen (Figure 2 c). At this stage we have not analyzed the intermolecular contributions to singlet relaxation.

Figure 3 compares field-dependent singlet relaxation decay rates (*T*_S_^−1^) as obtained by experiments on the degassed sample, and by simulation. A qualitative agreement (ca. 30 %) is obtained between the simulated relaxation rate constants and the experimental results. The simulated values of *T*_S_^−1^ have confidence limits of ±30 %, which derive mainly from uncertainties in estimates of the chemical shift tensors, the spin-rotation tensors, and the correlation times.

In summary, we have demonstrated a molecular prototype that displays a singlet decay time constant of more than one hour in a room-temperature liquid. We have identified the key relaxation mechanisms responsible for relaxation in this long-time regime and set up a computational methodology for predicting the singlet relaxation rates with good accuracy. The understanding of these mechanisms and the ability to predict singlet relaxation can drive the design of molecules possessing even longer-lived spin states. We anticipate that such systems will be useful for the transport and storage of nuclear hyperpolarization under ambient conditions. Since these molecular systems are amenable to chemical functionalization (unlike ^15^N_2_O and noble gases) we anticipate applications to molecular sensing and binding studies. Paramagnetic relaxation caused by dissolved oxygen remains significant but we expect to reduce this by suitable molecular design. We also plan to modify the system to achieve good water solubility. The measured singlet lifetime of more than 10 minutes even without degassing should permit applications in hyperpolarized NMR spectroscopy and MRI.

## Supporting Information

As a service to our authors and readers, this journal provides supporting information supplied by the authors. Such materials are peer reviewed and may be re-organized for online delivery, but are not copy-edited or typeset. Technical support issues arising from supporting information (other than missing files) should be addressed to the authors.

miscellaneous_information
