# Supplementary material for: A Nuclear Singlet Lifetime of More than One Hour in Room-Temperature Solution
Source: Angew Chem Int Ed Engl. 2015 Feb 4;54(12):3740–3. doi: 10.1002/anie.201411978 (PMC4497607; doi:10.1002/anie.201411978)
Supplement: Supplementary file 1 — miscellaneous_information [file anie0054-3740-sd1.pdf]

Angewandte Chemie
Eine Zeitschrift der Gesellschaft Deutscher Chemiker

Supporting Information

# A Nuclear Singlet Lifetime of More than One Hour in Room-Temperature Solution**

*Gabriele Stevanato, Joseph T. Hill-Cousins, Pär Håkansson, Soumya Singha Roy, Lynda J. Brown, Richard C. D. Brown, Giuseppe Pileio,* and Malcolm H. Levitt**

anie_201411978_sm_miscellaneous_information.pdf

**Computational Details**

The computational approach is described in detail elsewhere. In summary, the method involves the following steps: (1) A 2 ns classical molecular dynamics trajectory is computed using QM/MM (QM for the internal degrees of freedom of **I**, and MD for the intermolecular interactions); (2) The trajectory is sampled at 2 x $10^5$ points and spin interaction tensors computed (basis set 6-31G*, MP2 and DFT/B3PW91 for CSA and spin-rotation tensors respectively); (3) a time dependent spin Hamiltonian is generated by interpolating the interaction tensors in the spatial dimension (handling internal degrees of freedom by a Z-matrix); (4) the correlation functions of the fluctuating nuclear spin interactions are computed, including all cross-correlations; (5) Second-order perturbation theory is used to derive the relaxation rates from the spectral densities of the fluctuating spin interactions.

**Experimental**

$^{13}C_2$-**I** was dissolved in acetone-d6 at a concentration of 0.1M. For the degassed sample used in Figure 2a, 2b and Table 1, molecular oxygen was removed by 10 pump-thaw cycles. The singlet relaxation time constants ($T_S$) for $^{13}C_2$-**I** were measured by using the pulse sequence shown in Figure S1. For each transient, the sample was first equilibrated for almost one hour in

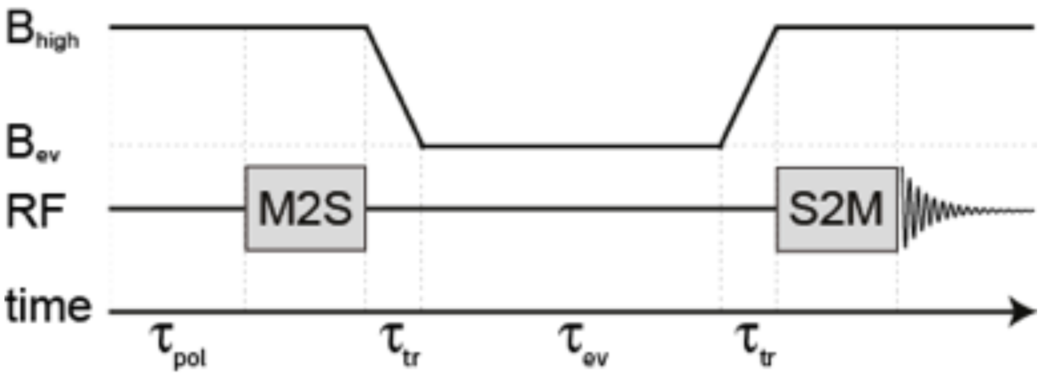


**Figure S1.** Sequence of events used to estimate $T_S$ at different magnetic fields $B_{ev}$. The M2S and S2M blocks are described in Ref.[4] and are here used with Δ = 4.62 ms, $n_1$=14 and $n_2$=7. The polarization time between successive transients was $\tau_{pol}$=3000 s to allow full thermal equilibration. The transport time $\tau_{tr}$ was between 0 to 8 s, depending on the evolution field $B_{ev}$. The time $\tau_{ev}$ spent at each field $B_{ev}$ was varied between 1 and 4500 s in 8 steps.

the high magnetic field (9.4 T) and then a magnetization-to-singlet (M2S) pulse sequence was run to convert longitudinal magnetization into long-lived singlet order. The sample was transported into a region of lower field, here indicated as the evolution field $B_{ev}$. The sample was left for a variable interval $\tau_{ev}$ at this field before to be returned to the high field where the singlet-to-magnetization (S2M) sequence was applied, and the NMR signal detected. The M2S and S2M pulse sequences are described in detail in Ref[1,2,3]. Sample transport was performed using the simple apparatus described in Ref.[1], which consists of a computer-controlled stepper motor that winds a string attached to the sample holder thus moving the sample along the main field axis. This procedure has the disadvantage that the steep profile of the magnet field may lead to a large difference in field between the top and bottom parts of the sample and hence to large error margins on the magnetic field value.

**References**

[1] G. Pileio, M. Carravetta, M. H. Levitt, *Proc. Natl. Acad. Sci. U.S.A.* **2010**, *107*, 17135–9.

[2] M. C. D. Tayler, M. H. Levitt, *Phys. Chem. Chem. Phys.* **2011**, *13*, 5556–60.

[3] G. Pileio, J. T. Hill-Cousins, S. Mitchell, I. Kuprov, L. J. Brown, R. C. D. Brown, M. H. Levitt, *J. Am. Chem. Soc.* **2012**, *134*, 17494–17497.

[4] G. Pileio, S. Bowen, C. Laustsen, M. C. D. Tayler, J. T. Hill-Cousins, L. J. Brown, R. C. D. Brown, J. H. Ardenkjaer-Larsen, M. H. Levitt, *J. Am. Chem. Soc.* **2013**, *135*, 5084–5088.
